# Prevalence and Hospital Admissions in Patients With Osteogenesis Imperfecta in The Netherlands: A Nationwide Registry Study

**DOI:** 10.3389/fendo.2022.869604

**Published:** 2022-04-25

**Authors:** Silvia Storoni, Sanne Treurniet, Alessandra Maugeri, Gerard Pals, Joost G. van den Aardweg, Stéphanie L. van der Pas, Mariet W. Elting, Peter Kloen, Dimitra Micha, Elisabeth Marelise W. Eekhoff

**Affiliations:** ^1^ Department of Internal Medicine, Section Endocrinology, Amsterdam Rare Bone Disease/Amsterdam Bone Center, Amsterdam University Medical Center, location VUmc, Amsterdam, Netherlands; ^2^ Department of Human Genetics, Amsterdam Movement Sciences, Amsterdam Rare Bone Disease/Amsterdam Bone Center, Amsterdam University Medical Center, location VUmc, Amsterdam, Netherlands; ^3^ Department of Respiratory Medicine, Amsterdam University Medical Center, location AMC, Amsterdam, Netherlands; ^4^ Department of Epidemiology and Data Science (EDS), Amsterdam University Medical Center, location VUmc, Amsterdam, Netherlands; ^5^ Department of Human Genetics, Amsterdam University Medical Center, location VUmc, Amsterdam, Netherlands; ^6^ Department of Orthopedic Surgery, Amsterdam Movement Sciences, Amsterdam Rare Bone Disease/Amsterdam Bone Center, Amsterdam University Medical Center, location AMC, Amsterdam, Netherlands

**Keywords:** osteogenesis imperfecta, morbidity, pathogenic variant, hospital admission, CBS registry

## Abstract

Osteogenesis Imperfecta (OI) is a complex disease caused by genetic alterations in production of collagen type I, and collagen-related proteins. Bone fragility is the most common patient issue, but extraskeletal complications also present an adverse factor in the quality of life and prognosis of patients with OI. However, still little is known about the morbidity and mortality of these patients. The objective of this paper is to determine and describe to what extent OI impacts patients’ life in terms of hospitalization and complications describing the incidence and prevalence of the Dutch cohort of OI patients and the characteristics of their hospital admissions. Information regarding OI patients and their hospital admission was extracted from the Statistics Netherlands Database and matched to the OI Genetics Database of Amsterdam UMC. Hospital admission data was available for 674 OI patients. This OI nationwide registry study shows that the life expectancy of OI patients is adversely affected by the disease. The median annual incidence risk of OI between 1992 and 2019 was 6.5 per 100,000 live births. Furthermore, patients with OI had a 2.9 times higher hospitalization rate compared to the general Dutch population. The highest hospitalization rate ratio of 8.4 was reported in the patient group between 0 and 19 years old. OI type and severity had impact on extraskeletal manifestations, which play a key role in the numerous hospital admissions. More awareness about the impact of OI on patients’ life is needed to improve and implement prevention and follow-up guidelines.

## Introduction

Osteogenesis Imperfecta (OI) is a connective tissue disorder caused by defects in genes encoding type I collagen (*COL1A1* and *COL1A2*) or proteins responsible for synthesis, posttranslational modification and transport of collagen type I. This results in aberrant bone formation.

OI has a relatively high phenotypic heterogeneity classified into five clinical types according to the Sillence classification (1, 2). OI type I is the most common and least severe form of OI leading to a limited number of fractures and bone deformities. Type II is perinatally lethal due to multiple severe fractures and pulmonary hypoplasia. OI type III is severe and characterized by multiple fractures, progressive deformations, kyphoscoliosis and short stature; patients are usually wheelchair dependent. Type IV OI can be mild to moderate with a limited number of fractures. Type V is characterized by mineralization of the interosseous membrane and hyperplastic callus formation. In the last two decades, new causative genes for OI have been discovered which stimulated the generation of the genetic classification system (types VI – XXI) (3). Considering that the clinical presentation of the newly discovered genes overlaps the Sillence types, we adhere to the latter classification in this study. It is of note that OI classification is largely based on the type and severity of skeletal manifestations and is limited informative about the variation in extraskeletal features which remain uncharted (4–6).

Although bone fragility is the most common patient issue, the disease also affects extraskeletal tissues in the form of dentinogenesis imperfecta, hearing loss and cardiopulmonary disease. These can severely affect OI patients’ quality of life and life expectancy due to a decline in function capabilities, well-being and subsequent morbidity (7–9).

Given the complexity of OI it remains challenging to determine multidisciplinary clinical guidelines and therapeutic regimes which adequately address the patients’ needs. The rare occurrence of the disease and differences in phenotype as well as the lack of information about the morbidity and mortality of OI patients, hinder the treating clinician in making informed decisions based on disease prognosis and impact of the disease on quality of life. To date, only a few studies describe mortality and cause of death in patients with OI reporting a normal lifespan and death from unrelated illnesses such as cardiovascular disease and malignancy for patients with milder types of OI (10, 11). In patients with a more severe OI type, it has already been shown that OI has a negative effect on survival (10–12). In 2016 Folkestad et al. described the mortality of a Danish nationwide OI population, where the all-cause mortality was nearly three times higher compared to that of an age- and gender- matched reference population. Patients with OI showed increased risk of death due to respiratory diseases, gastrointestinal disease, and trauma compared to the reference population. This study also described a median survival time of 72.4 years for male patients, compared to 81.9 in the reference population. The median survival time for females with OI was 77.4 years, compared to 84.5 years in the reference population (13). Vitale et al. reported a mortality rate of a pediatric OI cohort during hospitalization of 3% (14). Josep Darbà et al. reported a hospitalization incidence of 5.64 per 100,000 patients in a Spanish OI cohort between 2000 and 2017. They also analyzed the direct associated medical cost per hospital admission (15). The most recent study, by Kolovos et al., describes the characteristics of hospital admissions by patients with OI in the English National Health Service (NHS) in terms of incidence and costs of admissions (16). This scarcity of reported studies critically points to the requirement for more attention to OI morbidity and its impact on quality of life.

This is the first study to examine hospital admissions of OI patients in the Netherlands. The aim of this study is to describe the Dutch cohort of OI patients and the characteristics of their hospital admissions. By raising awareness about the impact of the disease on patients’ life, physicians can improve follow-up and therapeutic approaches to ameliorate their health and quality of life.

## Methods

### Study Participants

All patients genetically diagnosed with OI in the national reference center for the molecular diagnosis of OI in the Amsterdam UMC between December 1991 and April 2021 were eligible for inclusion in this study. Pathogenic OI variants were identified in *COL1A1, COL1A2, CRTAP, TMEM38B, IFITM5, CREB3L1, FKBP10, PLOD2, SP7, SERPINF1, P3H1, BMP1* and *PPIB* (3). The pathogenic variants in *SERPINH1* and *KDELR2* were excluded because these genetic variants had been found in the research setting and are not yet in the diagnostic database. In 20 patients no causative pathogenic variant for OI was found. However, protein analysis showed disruption and/or overmodification of collagen synthesis. Therefore, these 20 patients were included as well. In addition to their pathogenic variants, patients were divided into mild OI or severe OI. OI was classified as mild in case of OI Sillence type I and I/IV, and as severe in case of OI Sillence type II, III and IV. This classification is based on the data provided by the referring physician for the genetic analysis. This classification was not available for all patients (n = 418, 58%).

### Data Extraction

A cohort of 724 patients genetically diagnosed with OI between December 1991 and April 2021 were extracted from the Amsterdam UMC Genome Database based on the reported presence of a pathogenic variant(s). Statistics Netherlands (CBS) has anonymized health care data of the Dutch population which is available for research upon request by health authorities and academic institutions. A match between the Amsterdam UMC Genome Database cohort and the CBS-cohort was established in 93% (n=674) of the OI patients. For the matched patients we retrieved information on many aspects of hospitalization (available between 2013-2019) and, when applicable, age at time of death (available between 1995-2019).

### Data Analysis

Information regarding patients’ age, date of admission, type of admission, hospital type, admission duration, medical specialism, performed medical procedures, type of residence at the time of hospital admission and, if applicable, age at time of death was extracted from the Statistics Netherlands database. Two different age categorizations were used in this paper dictated by data availability. For the description of OI severity by age patients were allocated to the following seven age groups: 0 to 9, 10 to 17, 18 to 24, 25 to 39, 40 to 59, 60 to 74 and 75 years and older. For comparison with the Dutch population only broader age groups were available. Patients were categorized in five age groups: 0 to 19, 20 to 44, 45 to 64, 65 to 79 and 80 years or older. Data analysis is mainly descriptive. The birth incidence for OI was calculated between 1992 and 2019. Incidence rate ratios (IRR) were calculated for hospital admission by age category compared to the total Dutch population between 2013 and 2019 (mean n = 17,004,857). The numerator of the IRR was the mean of the total admissions per year (between 2013 and 2019), the denominator being the mean of the total OI population per year (between 2013 and 2019). This was also performed for each age category. Information regarding hospital admission numbers in the Dutch population was freely accessible *via* the Statistics Netherlands web page (www.cbs.nl).

Data are presented in absolute numbers, percentage and mean or median values with standard deviations. To ensure patient confidentiality, hospitalization information regarding patient groups lower than 10 is not always shown in the results. Groups with fewer than 10 patients are only shown when they cannot be directly traced back to a person. Statistical analyses were performed using IBM SPSS Statistics for Windows version 25 (IBM corporation, Armonk, NY, USA).

### Ethical Consideration

The Medical Ethics Review Committee (MERC) of the Amsterdam UMC (Amsterdam, The Netherlands) waived the need for ethics approval and the need to obtain consent for the analysis and publication of the retrospectively obtained and anonymized data for this non-intervention study (MERC study number 2021.0085).

## Results

**Figure 1:**
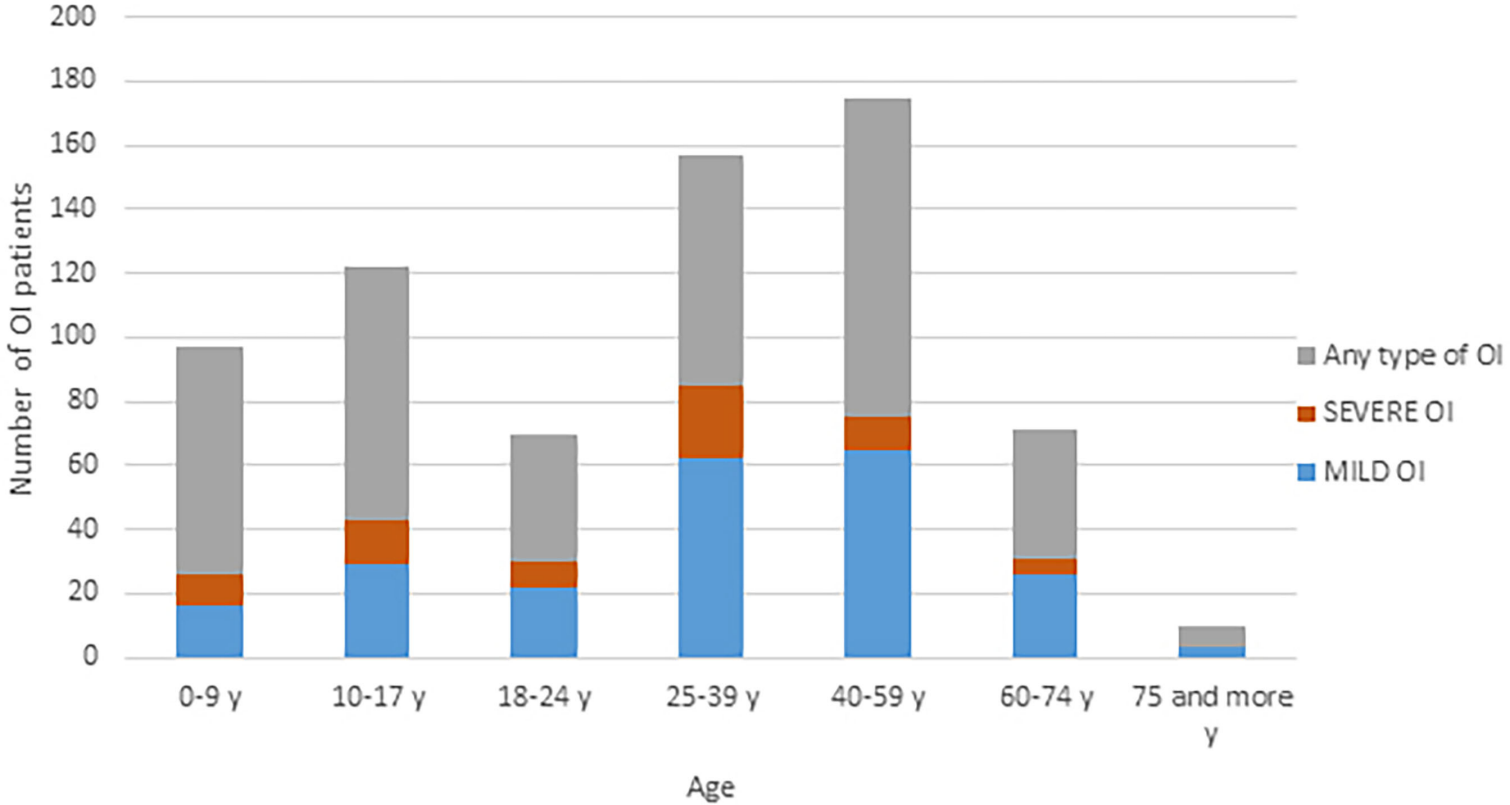
Age distribution of the Dutch OI population in 2021. OI, osteogenesis imperfecta; y, year.

We identified 724 patients genetically diagnosed with OI from the Amsterdam UMC Database. In addition to these patients, we identified 102 stillborn OI cases in which genetic analysis showed OI. These patients were assigned as OI type II. No further information about this group was available. The median annual incidence of OI between 1992 and 2019 was 6.5 (range 3 to 10.8) per 100,000 live births. Between 1995 and 2019 only 22 patients died. The causes of death could not be described as these subcategories contain less than 10 patients. Our cohort counts 724 patients, which represents approximately 85% of the OI patients. The estimated total number of OI patients in The Netherlands is around 850, with an estimated point prevalence 0.005% in the general population (April 2021). This estimated prevalence does not include the perinatally lethal cases of OI. **Figure 1** shows the age of the OI cohort in the Netherlands in 2021, as stratified by disease-severity. The highest number of OI patients is found in the age category of 40-59 years. 31% of the 724 patients were classified as mild OI, 11% as severe. A total of 58% of the patients could not be classified according to Sillence classification.

In total, 2225 hospital admissions of OI patients were registered in 479 patients between 2013 and 2019. Of the 674 CBS-matched patients, 195 (28.9%) patients were not admitted, 273 (40.5%) were admitted 1-3 times, 98 (14.5%) were admitted 4-6 times, 58 (8.6%) were admitted 7-10 times and 50 (7.4%) were admitted more than 11 times (mean: 3.30 median: 2.0 std dev: 4.55).The average incidence rate ratio (IRR) for admission of OI patients compared to the reference population in the Netherlands is 2.9. **Table 1** shows the IRR by age category for OI patients compared to the reference population. All age categories showed a higher IRR for admission for OI patients compared to the reference population, with the highest admission rate in the lowest age category (0 to 19 years). In the older age categories, the IRR decreases. The IRR was not available for the age category of 80 years and older due to the low number of OI patients.

**Table 1:** Number of hospitalizations and IRR in the reference population and the OI cohort between 2013 and 2019 in the Netherlands.

	*Age intervals*						
	*0-19 y*	*20-44 y*		*45-64 y*	*65-79 y*	*80-100 y*	*Total*
Mean admission per year in the Dutch population (2013-2019)	415,856	610,732		940,815	966,066	384,341	3,317,714
Mean Dutch population per year (2013-2019)	3,826,143	5,320,426		4,779,857	2,329,143	749,426	17,004,857
Incidence rate (per year):	0.11	0.11		0.20	0.42	0.51	0.19
Mean admission per year in OI Dutch cohort (2013-2019)	215	67		49	16	x	351
Mean OI Dutch cohort per year (2013-2019)	235	236		126	26	x	625
Incidence rate (per year):	0.92	0.29		0.39	0.62	x	0.56
IRR	8.4	2.6		2.0	1.5	x	2.9

Y, year; OI, Osteogenesis Imperfecta; IRR, Incidence rate ratio; x, not available.

**Table 2** shows the characteristics of the OI admissions in the Netherlands. Seventy seven percent of the total hospital admissions (n=2225) was for planned care. Of these, 64.5% of the patients were referred for daytime admission and 35.5% for inpatient admission. The mean length of stay for a clinical admission was 4.50 days (median: 2, std dev: 8.82, range: 184), the mean length of hospital stay for patients with mild OI was 3.59 days, and for patients with severe OI was 4.09 days (**Supplement 1**). The mean length of hospital stay was 5.05 days for patients with no available Sillence classification (median: 2, std dev: 10.99, range 183).

**Table 2:** Hospital admission characteristics.

		OI severity			Type of admission	
Admission urgency		Mild n (%)	Severe n (%)	Missing n (%)	Daytimen (%)	Clinical n (%)
Total	2225 (100%)	654 (100%)	311 (100%)	1260 (100%)	1434 (100%)	791 (100%)
Acute	507 (22.8%)	143 (21.9%)	49 (15.8%)	315 (25.0%)	45 (3.1%)	462 (58.4%)
Non-acute	1718 (77.2%)	511 (81.1%)	262 (84.2%)	945 (75.0%)	1389 (96.9%)	329 (41.6%)
**Hospital type**						
Total	2225 (100%)	654 (100%)	311 (100%)	1260 (100%)	1434 (100%)	791 (100%)
UMC	974 (43.8%)	267 (40.8%)	198 (63.7%)	509 (40.4%)	697 (48.6%)	277 (35.0%)
Topclinical hospital	806 (36.2%)	251 (38.4%)	67 (21.5%)	488 (38.7%)	470 (32.8%)	336 (42.5%)
General hospital	445 (20.0%)	136 (20.8%)	46 (14.8%)	263 (20.9%)	267 (18.6%)	178 (22.5%)
**Admission duration**						
Total	2225 (100%)	654 (100%)	311 (100%)	1260 (100%)	1434 (100%)	791 (100%)
<24 hour	1448 (65.1%)	421 (64.4%)	214 (68.8%)	813 (64.5%)	1428 (99.6%)	20 (2.5%)
1-3 days	510 (22.9%)	163 (24.9%)	58 (18.6%)	289 (22.9%)	6 (0.4%)	504 (63.7%)
4-10 days	190 (8.5%)	52 (8.0%)	33 (10.6%)	105 (8.3%)	0	190 (24.0%)
>10 days	77 (3.5%)	18 (2.7%)	6 (2.0%)	53 (4.3%)	0	77 (9.8%)
**Age groups**						
Total	2223 (100%)	654 (100%)	311 (100%)	1258 (100%)	1434 (100%)	789 (100%)
0 Y	134 (6.0%)	19 (2.9%)	15 (4.8%)	100 (7.9%)	41 (2.9%)	93 (11.8%)
1 - 19 Y	1305 (58.7%)	342 (52.3%)	202 (65.0%)	753 (59.9%)	1024 (71.4%)	281 (35.6%)
20 - 44 Y	432 (19.5%)	162 (24.8%)	59 (19%)	214 (17.0%)	203 (14.2%)	229 (29.0%)
45 – 64 Y	272 (12.2%)	100 (15.3%)	34 (10.9%)	141 (11.2%)	135 (9.4%)	137 (17.3%)
65 – 79 Y	80 (3.6%)	31 (4.7%)	1 (0.3%)	50 (4.0%)	31 (2.1%)	49 (6.2%)
**Medical specialty**						
Total	2225 (100%)	654 (100%)	311 (100%)	1260 (100%)	1434 (100%)	791 (100%
Pediatrics	911 (40.9%)	201 (30.7%)	147 (47.2%)	563 (44.7%)	796 (55.5%)	115 (14.5%)
Surgery	286 (12.9%)	122 (18.7%)	14 (4.5%)	150 (11.9%)	123 (8.6%)	163 (20.6%)
Orthopedic surgery	440 (19.8%)	138 (21.1%)	100 (32.2%)	202 (16.0%)	172 (12.0%)	268 (33.9%)
Internal medicine	121 (5.4%)	38 (5.8%)	23 (7.4%)	60 (4.8%)	89 (6.2%)	32 (4.0%)
Neurology	53 (2.4%)	10 (1.5%)	3 (1.0%)	40 (3.2%)	19 (1.3%)	34 (4.3%)
Otolaryngology	80 (3.6%)	26 (3.9%)	4 (1.3%)	50 (3.9%)	56 (3.9%)	24 (3.0%)
Ophthalmology	26 (1.2%)	8 (1.2%)	3 (1.0%)	15 (1.2%)	*	*
Gynecology	85 (3.8%)	35 (5.4%)	1 (0.3%)	49 (3.9%)	17 (1.2%)	68 (8.6%)
Gastroenterology	91 (4.1%)	21 (3.2%)	2 (0.6%)	68 (5.4%)	69 (4.8%)	22 (2.8%)
Cardiology	34 (1.5%)	19 (2.9%)	0	15 (1.2%)	18 (1.3%)	16 (2.0%)
Pulmonology	30 (1.3%)	17 (2.6%)	3 (1.0%)	10 (0.8%)	10 (0.7%)	20 (2.5%)
Urology	26 (1.2%)	9 (1.4%)	2 (0.6%)	15 (1.2%)	14 (1.0%)	12 (1.5%)
Other	42 (1.9%)	10 (1.5%)	9 (2.9%)	23 (1.8%)	*	*

UMC, University Medical Center; Y, year; Topclinical hospital: 27 affiliated large teaching hospitals in The Netherlands. * numbers are not shown due to low patient numbers which may compromise patient privacy.

**Figure 2:**
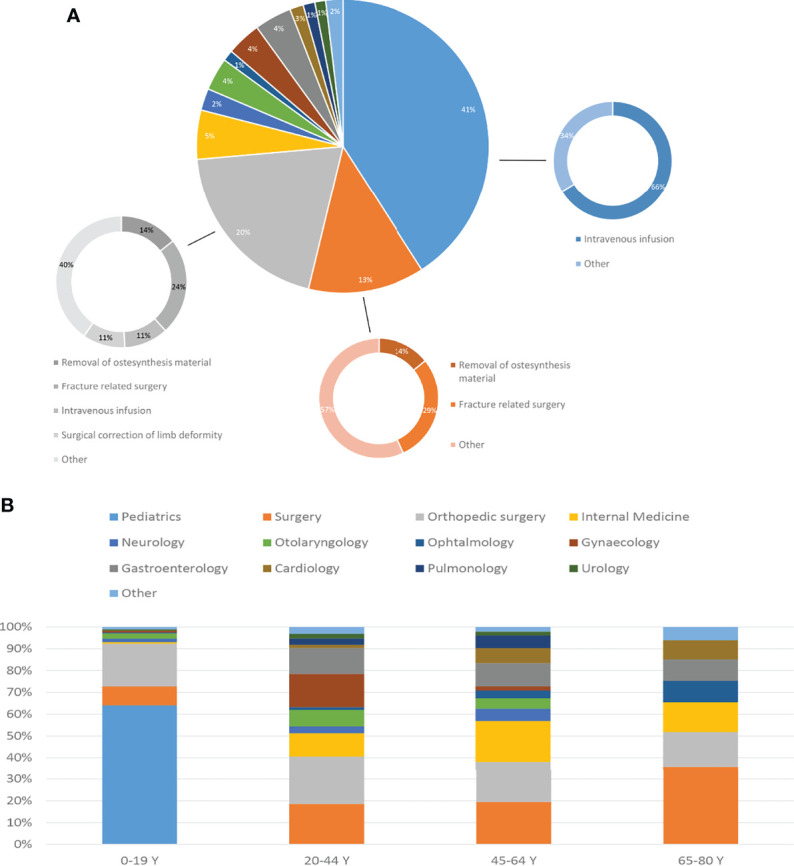
**(A)** Distribution of hospital admissions across medical specialties. For pediatrics, surgery and orthopedic surgery a further subdivision provides the reason of admission. **(B)** Age of admissions by medical specialty.

The vast majority (95%) of OI patients admitted to the hospital were living independently in their own residence. Most hospital admissions occurred in the youngest age group of 0-19 years in the pediatric ward (**Figure 2**). During the admissions in the youngest age group, the most commonly performed medical procedures were intravenous infusions, which most likely involved bisphosphonate treatment. In the older age categories, the admission percentage for the two main specialties: surgery and orthopedic surgery, remained nearly the same. An admission for an extraskeletal problem of OI was most frequent to internal medicine, neurology, gynecology, otolaryngology, ophthalmology, cardiology and pulmonology.

## Discussion

OI is a complex disease affecting collagen type I. It is characterized by multiple bone fractures and subsequent skeletal deformity. There are also extraskeletal symptoms that can play an important role in the life and prognosis of OI patients. Not much is known about the morbidity and mortality of OI patients, and to what extent the disease impacts their life in terms of hospitalization. The aim of this study was to adequately describe the Dutch cohort of OI patients and the characteristics of their hospital admissions.

This is the first study describing the characteristics of OI patients with regard to birth incidence and hospitalization in the Netherlands. The median annual birth incidence of OI between 1992 and 2019 was 6.5 per 100,000 live births. Compared to previous studies in Spain (10.14 per 100,000) and Denmark (15 per 100,000) this was relatively low (13, 15). Nowadays, 8.1% of the OI population in the Netherlands is over 65 years old, which is considerably lower than the percentage of people over 65 years old in the general population (20%) (**Supplement 2**) (17). Patients with OI in The Netherlands have a 2.9 times higher risk of hospitalization than the general population. This relative risk peaks in the youngest age category of 0 to 19 years old, where 8.4 times as much hospital admissions are observed as compared to the general population. This difference can probably be attributed to two factors: 1) most OI patients experience the highest fracture rate during their childhood and often require hospitalization; 2) treatment with bisphosphonates is most successful at pediatric age and is generally performed during daytime hospitalization.

Extraskeletal complications can also lead to hospitalizations, with gastroenterology, otolaryngology, ophthalmology and pulmonology being the most common. An admission to gastroenterology was in 26% of patient for a colonoscopy. In the young age group, pediatrics was the most frequently involved specialism, followed by general surgery and orthopedics. Older age groups had an increasing number of extraskeletal complications. These involved mostly clinical admissions to otorhinolaryngology, gastroenterology, cardiology and pulmonology. In the age group between 20 and 44 there was a considerable part of admissions to gynecology (**Supplement 3**). In the different age groups, most hospitalizations were noted for general surgery and orthopedics whereas in the younger age groups this also included pediatrics. This can be expected in the OI population considering the high bone fragility both at pediatric and adult phase; OI bone is known to have a lower capacity for energy absorption due to lower strength and elasticity, and can therefore more easily lead to fractures (18, 19). Unfortunately, due to lack of data, it was not possible to compare the incidence of single-specialist hospitalizations between the OI population and the general population. Regarding the hospital type, this study showed that severe OI patients were predominantly admitted to a University Medical Center (UMCs) (63.7%), as compared to 40.8% of the mild OI patients who were admitted to UMCs and 38.4% who were seen in Topclinical hospitals (large teaching hospitals). Furthermore, in the group of patients with the most severe form of OI, the proportion of patients between 1 and 19 years of age was 65%, the proportion of patients between 20 and 44 years of age was 19%, patients between 45 and 64 years was 10.9%, and patients between 65 and 79 years of age was 0.3%. The latter has a much younger population compared to mild OI patients, with 52.3% of patients between 1 and 19 years of age, the proportion of patients between 20 and 44 years old 24.8%, between 45 and 64 15.3%, and between 65 and 79 was 4.7%. OI severity is also associated with differences in hospital admissions related to clinical services for extraskeletal problems. Mild OI patients showed higher percentage of admissions to the otorhinolaryngology, ophthalmology, gynecology, cardiology and pulmonology specialisms compared to severe OI patients. The overall low number of hospital admissions to otolaryngology, ophthalmology, cardiology and urology can be explained by the fact that much of this type of care is provided on outpatient basis. Outpatient care data was not available for this study.

This study is limited by various factors. Our database does not contain all OI patients in The Netherlands. It is estimated that our database contains an estimated 85% of Dutch OI patients. Not all clinically diagnosed patients have been tested with the current NGS panel for OI. Patients with negative test results or with test results obtained in research setting (e.g. during discovery of new OI genes) are also not in the cohort described herein. Another limitation is the lack of information about Sillence classification. For the Sillence classification we relied on the information provided by a great many healthcare professionals across The Netherlands requesting the molecular diagnosis. Misclassification of patients cannot be ruled out considering that classification according to Sillence is difficult and requires a lot of experience. The Sillence classification data was missing in 58% of the patients. Unfortunately due to strict privacy rules from the CBS it was not allowed to publish information regarding causes and age of death of patients with OI. We also had no further information regarding the intravenous infusion as we could not describe the type of bisphosphonates used and the duration of treatment. There are also numerous strengths. This is the first study describing the Dutch cohort of OI patients and the characteristics of their hospital admissions. This study highlights interesting differences between age groups, severity of the disease and extraskeletal manifestations.

In conclusion, this study shows that life expectancy of OI patients is adversely affected by the disease. The percentage of patients over 65 in the OI population is 2.5 times smaller than in the general Dutch population. Furthermore, this study shows that patients with OI are admitted 2.9 times more often to a hospital compared to the general population. The highest admission rate ratio of 8.4 was identified in the patients’ group between 0 and 19 years old. This study also highlights the role of the type and severity of the disease on extraskeletal manifestations, which play a key role in the numerous hospital admissions. The differences in frequency and type of hospital admissions based on OI severity provide a concrete representation of the disease effect and impact of OI on quality of life. With this nationwide registry study of OI hospitalization in the Netherlands we hope to aid clinical specialists in developing and implementing guidelines that can improve the quality of life in OI patients.

## Data Availability Statement

The original contributions presented in the study are included in the article/**Supplementary Material**. Further inquiries can be directed to the corresponding author.

## Author Contributions

All authors listed have made a substantial, direct, and intellectual contribution to the work, and approved it for publication.

## Conflict of Interest

The authors declare that the research was conducted in the absence of any commercial or financial relationships that could be construed as a potential conflict of interest.

The reviewer OS declared a past co-authorship with one of the authors EE to the handling editor.

## Publisher’s Note

All claims expressed in this article are solely those of the authors and do not necessarily represent those of their affiliated organizations, or those of the publisher, the editors and the reviewers. Any product that may be evaluated in this article, or claim that may be made by its manufacturer, is not guaranteed or endorsed by the publisher.
