# Supplementary material for: Prevalence and Hospital Admissions in Patients With Osteogenesis Imperfecta in The Netherlands: A Nationwide Registry Study
Source: Front Endocrinol (Lausanne). 2022 Apr 25;13:869604. doi: 10.3389/fendo.2022.869604 (PMC9082351; doi:10.3389/fendo.2022.869604)
Supplement: Supplementary file 1 [file Table_1.docx]

**Supplementary material**

| **Total clinical admissions** (791) | **Mild OI** (236) | **Severe OI** (98) | **Unknown Sillence classification** (457) |
| --- | --- | --- | --- |
| Mean: 4.50 | Mean: 3.59 | Mean: 4.09 | Mean: 5.05 |
| Median: 2 | Median: 2 | Median: 2.5 | Median: 2 |
| Std. Deviation: 8.816 | Std. Deviation: 4.220 | Std. Deviation: 4.245 | Std. Deviation: 10.994 |
| Range: 184 | Range: 27 | Range: 31 | Range: 184 |

**Supplement 1**. Mean hospital admission stay in days.

| **2021** | **0-19 Y** | **20-39 Y** | **40-64 Y** | **65-79 Y** | **>80 Y** |
| --- | --- | --- | --- | --- | --- |
| Dutch population | 21% | 25% | 34% | 15% | 5% |
| OI population | 33.6% | 28.5% | 29.8% | 8.0% | 0.1% |

**Supplement 2.** Age distribution across the general Dutch and OI population that was alive in 2021. Y = years

| **Age** | **0 Y** | **1-19 Y** | **20-44 Y** | **45-64 Y** | **65-79 Y** | **>80 Y** |
| --- | --- | --- | --- | --- | --- | --- |
| Total | 6.02% | 58.02% | 19.64% | 12.45% | 3.76% | 0.09% |
| **Age** | **0 Y** (100%) | **1-19 Y** (100%) | **20-44 Y** (100%) | **45-64 Y** (100%) | **65-79 Y** (100%) | **>80 Y** |
| Pediatrics | 79.85% | 62.42% | 0 | 0 | 0 | X |
| Surgery | 3.73% | 9.16% | 18.54% | 19.42% | 35.80% | X |
| Orthopedic surgery | 3.73% | 21.27% | 21.97% | 18.71% | 16.05% | X |
| Internal medicine | 0 | 0.78% | 10.76% | 18.71% | 13.58% | X |
| Neurology | 3.73% | 1.39% | 3.20% | 5.76% | 0 | X |
| Laryngology | 0 | 2.64% | 7.55% | 4.68% | 0 | X |
| Ophthalmology | 0 | 0.39% | 1.14% | 3.60% | 9.88% | X |
| Gynaecology | 8.96% | 0 | 15.33% | 1.80% | 0 | X |
| Gastroenterology | 0 | 0 | 11.90% | 10.79% | 9.88% | X |
| Cardiology | 0 | 0 | 1.37% | 6.83% | 8.64% | X |
| Pulmonology | 0 | 0 | 2.97% | 5.76% | 0 | X |
| Urology | 0 | 0.70% | 2.06% | 1.80% | 0 | X |
| Other | 0 | 1.24% | 3.20% | 2.16% | 6.17% | X |

**Supplement 3.** Age by admission per medical specialism. Y = years
